# Mitochondrion-associated protein peroxiredoxin 3 promotes benign prostatic hyperplasia through autophagy suppression and pyroptosis activation

**DOI:** 10.18632/oncotarget.17927

**Published:** 2017-05-17

**Authors:** Min-Yao Jiang, Zhao-Dong Han, Wenjiao Li, Fei Yue, Jianheng Ye, Bowei Li, Zhiduan Cai, Jian-Ming Lu, Weimin Dong, Xianhan Jiang, Weide Zhong, Huichan He, Leyuan Liu

**Affiliations:** ^1^ Department of Urology, Guangdong Key Laboratory of Clinical Molecular Medicine and Diagnostics, Guangzhou First People's Hospital, Guangzhou Medical University, Guangzhou, China; ^2^ Institute of Biosciences and Technology, Texas A&M University, Houston, Texas, USA; ^3^ Department of Urology, The Fifth Affiliated Hospital of Guangzhou Medical University, Guangzhou, Guangdong Province, China; ^4^ Department of Molecular and Cellular Medicine, College of Medicine, Texas A&M University, College Station, Texas, USA

**Keywords:** autophagy, benign prostatic hyperplasia, BPH, caspase 1, LC3

## Abstract

Benign prostatic hyperplasia (BPH) is one of the most common diseases in the senior men and age plays an important role in the initiation and development of BPH. Mammalian cells primarily use the autophagy-lysosome system to degrade misfolded/aggregated proteins and dysfunctional organelles such as mitochondria and suppress pyroptosis, a type of cell death that stimulates inflammatory responses and growth of other cells around. Peroxiredoxin 3 (PRDX3) is the only mitochondrion-associated member of peroxiredoxin family enzymes that exert their protective antioxidant role in cells through their peroxidase activity. We hypothesized that PRDX3 may inhibit autophagy to activate pyroptosis to induce growth of prostatic epithelial cells. Here we show that PRDX3 maintained the integrity of mitochondria and its depletion led to an enhancement of oxidative stresses. PRDX3-associated and PRDX3-free mitochondria co-existed in the same cells. PRDX3 expressed at higher levels in prostatic epithelial cells in prostate tissues from BPH patients and BPH-representative cell line than in prostate tissues from healthy donors and a cell line representing normal epithelial cells. PRDX3 suppressed autophagy flux and activated pyroptosis to induce inflammatory responses and stimulate the over-growth of prostate tissues. Therefore, higher levels of PDRX3 in prostatic epithelial cells may promote the initiation and development of BPH through autophagy inhibition and pyroptosis activation.

## INTRODUCTION

*Benign* prostatic hyperplasia (BPH) is a condition in men whose prostate gland is enlarged because of the progressive hyperplasia of stromal and glandular prostatic cells and not cancerous [1]. It is the most common prostate problem for senior males and the main cause of urological dysfunction of middle-aged to senior men. The autopsy results from different geographical regions show that the incidence of histological BPH increases with ages [2]. Along with the increases of average lifespans due to the improvement of living standards and health conditions, incidences of BPH increase significantly. Although, α-blockers and 5α reductase inhibitors have achieved some beneficial outcomes in the treatment of BPH in recent years, both types of drugs exhibit different efficacies among different patients but certain side effects, and need to be used for long term but are not able to cure the disease [3]. Therefore, it is of great significance to promote the researches leading to BPH prevention.

Mammalian cells primarily use the autophagy-lysosome system to degrade dysfunctional organelles, misfolded/aggregated proteins and other macromolecules [4]. Defects in autophagy lead to an enhancement of oxidative stress [5, 6] or lysosomal rupture [7] which in turn activates NLRP3 inflammasomes that result in direct activation of caspase-1 [8]. Activation of caspase-1 eventually induces pyroptosis, an inflammatory form of cell death [9]. The release of immunogenic danger signals or danger-associated molecular patterns (DAMPs) from pyroptotic cells can fuel pro-inflammatory cascades that promote the mortality of host structural, hematopoietic and immune-competent cells [10, 11]. Inflammation induces compensative growth of both epithelial and stromal cells and enlargement of prostate [12, 13]. Therefore, autophagy may directly regulate BPH.

Peroxiredoxin 3 (PRDX3) is the only mitochondrion-associated member of peroxiredoxin family enzymes that exert their protective antioxidant role in cells through their peroxidase activity [14]. It was reported that PRDX3 is overexpressed in prostate cancer and promotes cancer cell survival by protecting cells from oxidative stress [15–17]. Since oxidative stress also induces autophagy [18], high levels of PRDX3 may lead to autophagy inhibition through oxidative stress suppression. We hypothesized that PRDX3 may inhibit autophagy to affect BPH. Here, we found the levels of PRDX3 protein in prostate tissues from BPH patients were significantly higher than those from healthy donors. PRDX3 was associated with mitochondria and its silence led to an enhancement of oxidative stress. High levels of PRDX3 led to an inhibition of autophagy flux and an activation of pyroptosis. Therefore, up-regulation of PRDX3 in prostatic tissues may promote BPH.

## RESULTS

### The expression of PRDX3 protein increases in epithelial cells of prostatic glands from BPH patients

**Figure 1:**
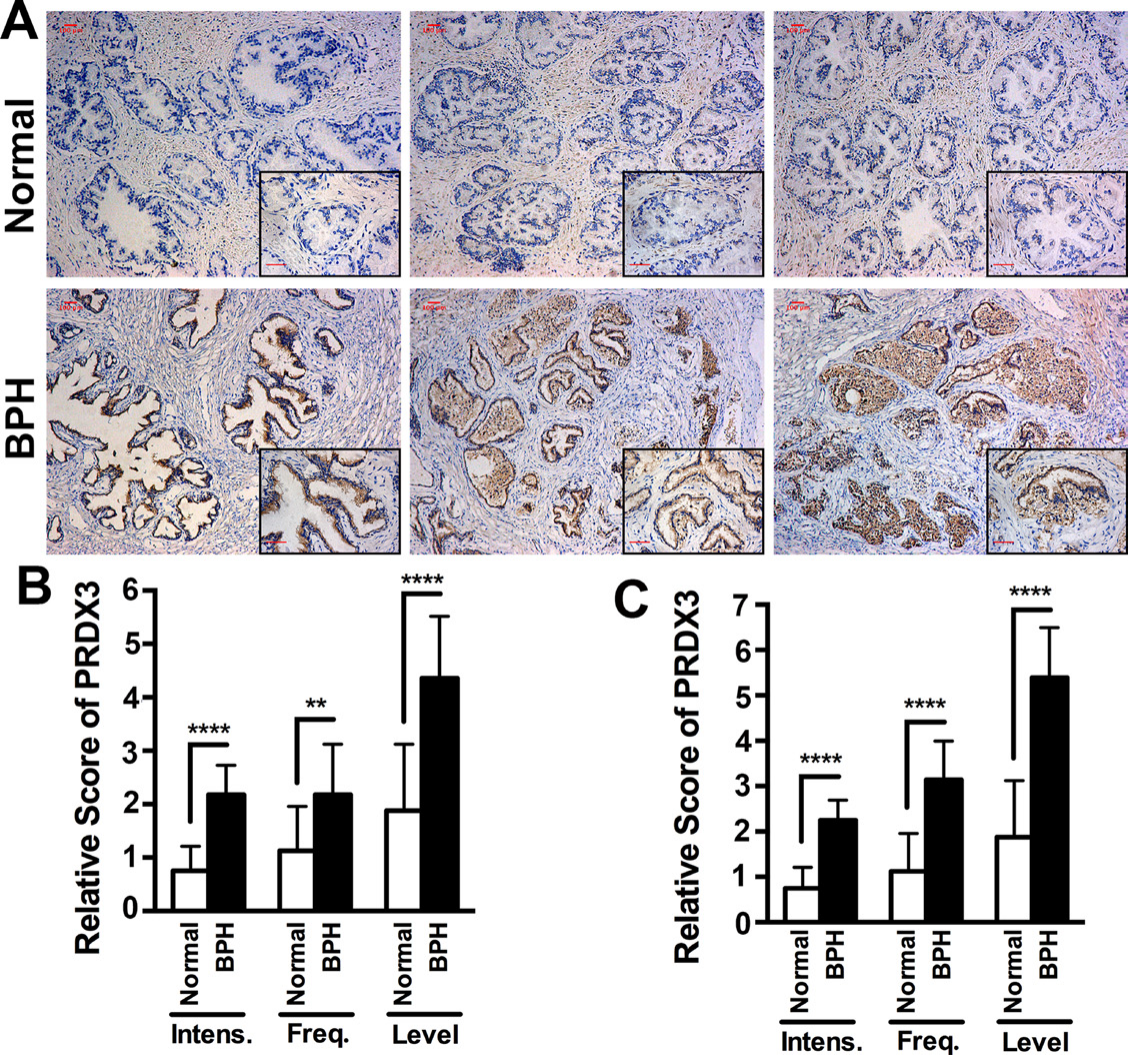
The expression of PRDX3 protein in human prostatic tissues (**A**) Representative images showing the immunostaining patterns of PRDX3 in prostatic tissues from healthy donors or BPH patients. The insets in black boxes are enlarged images. Bar = 100 μm. (**B**, **C**) Comparison of the levels of PRDX3 in total prostate tissues (B) or epithelial cells (C) from normal healthy donors and BPH patients. Bars represent the relative score of PRDX3 intensity (Intens), frequency (Freq) and level. **P ≤ 0.01; and ****P ≤ 0.0001.

It was reported that levels of PRDX3 protein are higher in prostate tumor tissues than in their adjacent normal prostate tissues or disease-free normal prostate tissues, suggesting a potential role of PRDX3 in prostate adenocarcinomas [15–17]. To investigate the role of PRDX3 in the development of BPH, we collected normal prostate tissues from 8 healthy donors and BPH tissues from 28 patients to conduct histoimmunochemical staining using an anti-PDRX3 antibody. We found that BPH exhibited significantly higher levels of PRDX3 than in normal prostrate tissues (Figure 1A, Table 1). The relative intensity of PRDX3 staining, the frequency of cells with positive signals and final scores of PRDX3 expression in BPH tissues, especially in epithelial cells, were significantly higher than in normal prostate tissues (Figure 1B, 1C). Further examination of the relationship of PRDX3 expression with the ages, serum PSA levels and prostate gland volumes among BPH patients did not found any difference (Table 1). Therefore, PRDX3 protein is overexpressed in the epithelial cells of prostate glands of BPH patients.

**Table 1:** PRDX3 levels and clinico-pathological characteristics

Clinical features	Case No.	$\bar{X}\pm s$	P
**Normal prostate tissues**	8	1.88 ± 1.25	< 0.001
**BPH**	28	4.36 ± 1.16	
**Age (years)**			
** ≤ 65**	5	3.80 ± 1.30	0.244
** > 65**	23	4.48 ± 1.12	
** Serum PSA levels (ng/ml)**			
** < 4**	5	4.40 ± 1.34	0.929
** ≥ 4**	23	4.35 ± 1.15	
**Prostate gland volume (ml)**			
** < 75**	16	4.06 ± 0.93	0.123
** ≥ 75**	12	4.75 ± 1.36	

* $\bar{X}\pm s$, the mean ± standard deviation of PRDX3 levels.

### The expression of PRDX3 protein in cultured prostate cells is similarly increased and associated with levels of autophagy flux

**Figure 2:**
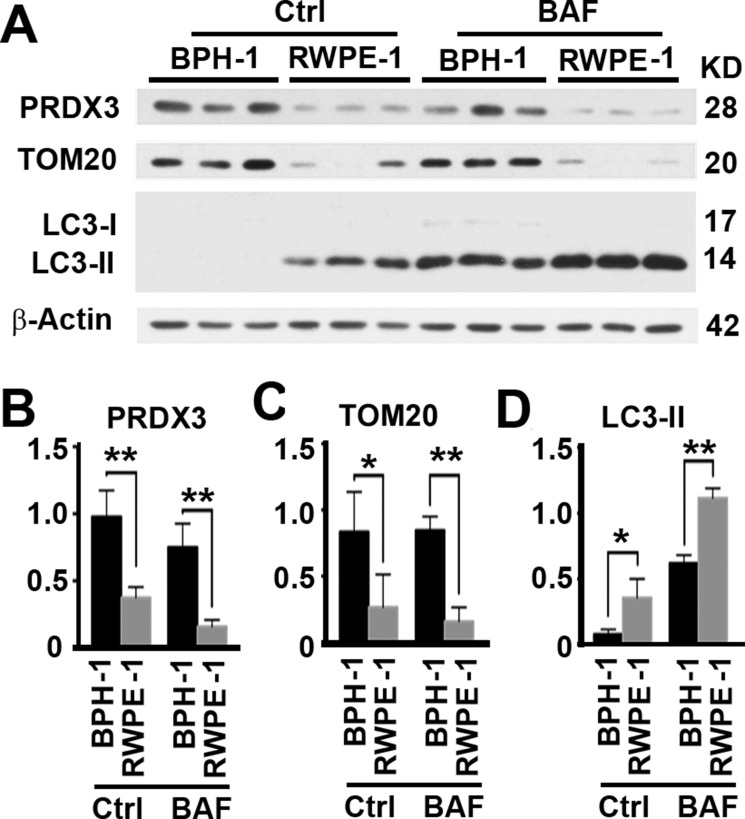
PRDX3 expression and its association with autophagy flux in cultured prostate cells (**A**) Representative immunoblot showing the levels of PRDX3, TOM20 and LC3-II in in lysates prepared from three different cultures of BPH-1 and RWPE-1 cells in the absence (Ctrl) or presence of bafilomycin A1 (BAF). (**B**–**D**) The quantification of the relative levels of PRDX3 (B), TOM20 (C) and LC3-II (D) to β-Actin as shown in (A). Data are mean and standard deviation of three repeats and differences are tested with Student's *T*-test. *P ≤ 0.05; **P ≤ 0.01.

Cell line BPH-1 represents cells originating from human benign prostate hyperplasia and RWPE-1 represents normal adult human prostatic cells, respectively. Both cell lines are epithelial. In consistent with results from human tissues, levels of PRDX3 protein were significantly higher in BPH-1 than in normal prostate cells (Figure 2A, 2B). In addition, total mitochondrial mass represented by the levels of TOM20 protein was more (Figure 2A, 2C). Levels of LC3-II represent a balance between synthesized and degraded autophagosomes. In order to measure autophagy flux, we treated cells with lysosomal inhibitor bafilomycin A1 to compare the amounts of autophagosomes accumulated during the same period of time. We found that levels of LC3-II in the presence of bafilomycin A1 were lower in BPH-1 than in normal prostate cells (Figure 2A, 2D), suggesting PRDX3 inhibits autophagy flux. Therefore, PRDX3 may impair autophagy flux and induce accumulation of mitochondria in BPH-1.

### Suppression of mitochondrion-associated PRDX3 leads to enhancement of oxidative stress in BPH-1 cells

**Figure 3:**
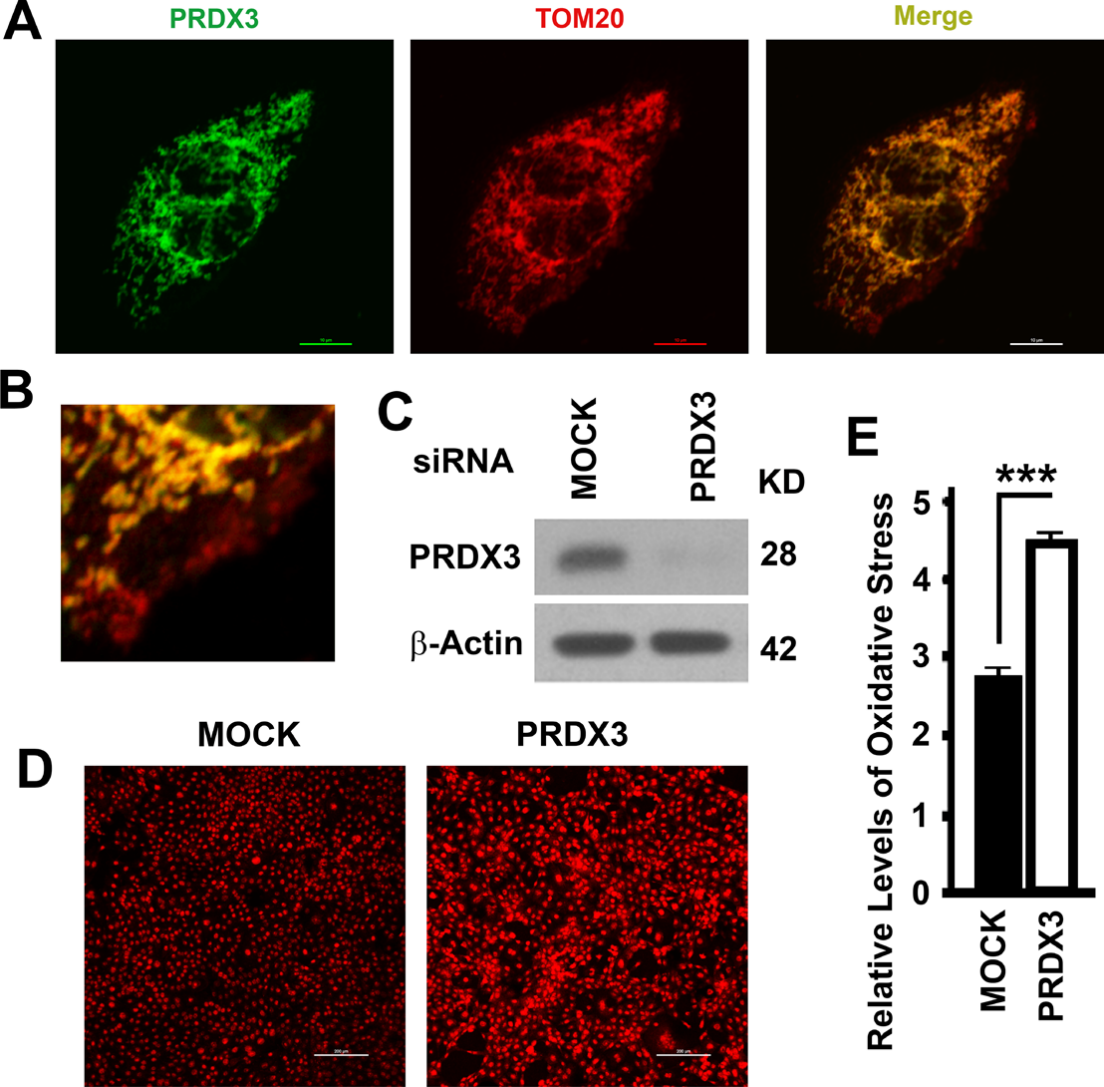
Mitochondrial association and impacts on oxidative stress of PRDX3 (**A**) Representative images showing the colocalization of PRDX3 (green) with TOM20 (red) in BPH-1 cells. Bar = 10 μm. (**B**) A image showing a part of the merge showing in (A). (**C**) A representative immunoblot showing the levels of PRDX3 in BPH-1 cells treated with random (MOCK) or PRDX3-specific siRNA (PRDX3). (**D**, **E**) Representative images (D) and quantification (E) oxidative stress as indicated by the intensities of red fluorescence after staining with dihydroethidine hydrochloride. Bar = 200 μm. Data are mean and standard deviation of three repeats and differences are tested with Student's *T*-test. ***P ≤ 0.001.

PRDX3 was reported to be a mitochondrion-associated protein [19]. We confirmed the mitochondrial colocalization of PRDX3 with mitochondrial marker TOM20 in BPH-1 cells (Figure 3A). However, there were mitochondria stained with TOM20 which were devoid of PRDX3 (red signal, Figure 3B), indicating an accumulation of PRDX3-free mitochondria. To investigate the impact of PRDX3 on mitochondria, we selected a PRDX3-specific siRNA and efficiently suppressed the expression of PRDX3 in BPH-1 cells (Figure. 3C). PRDX3-deficient BPH-1 cells exhibited higher levels of oxidative stress than the control (Figure 3D, 3E). Therefore, PDRX3 suppresses mitochondrial oxidative stress.

### PRDX3 inhibits autophagy flux by reducing levels of PI3KCIII

**Figure 4:**
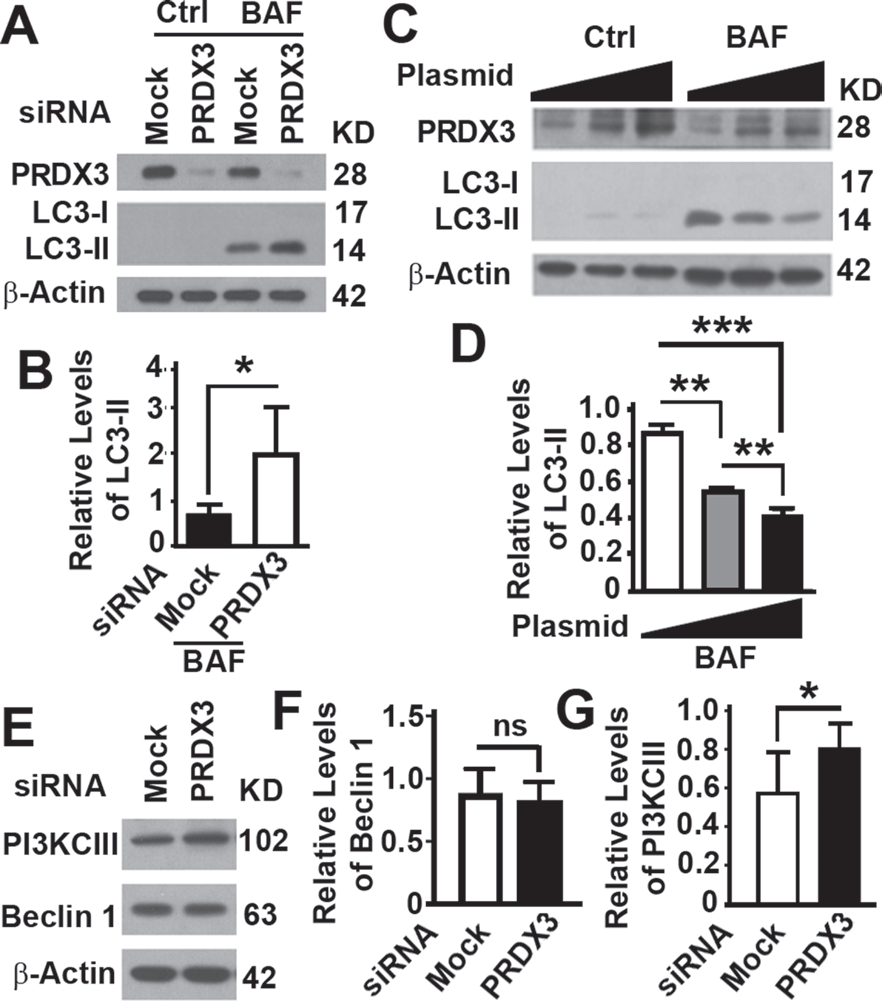
Impacts of PRDX3 protein on autophagy flux (**A**–**D**) Representative immunoblot (A, C) and quantification (B, D) showing the levels of LC3-II in BPH-1 cells treated with random (MOCK) or PRDX3-specific siRNA (PRDX3) (A, B) or RWPE-1 cells transiently expressing different amount of PRDX3 (C, D) in the absence (Ctrl) or presence of bafilomycin A1 (BAF). Data are mean and standard deviation of three repeats and differences are tested with Student's *T*-test. *P ≤ 0.05; **P ≤ 0.01; ***P ≤ 0.001. (**E**–**G**) Representative immunoblot (E) and quantification (F, G) showing the levels of Beclin 1 (F) and PI3KCIII (G) in BPH-1 cells treated with random (MOCK) or PRDX3-specific siRNA (PRDX3). Ns, not significant; *P ≤ 0.05.

To understand the mechanism by which PRDX3 promote BPH, we tested the role of PRDX3 in the autophagy regulation. Reducing the expression of PRDX3 in BPH-1 cells with high levels of PRDX3 led to an enhancement of autophagy flux as indicated by the relative levels of LC3-II in the presence of bafilomycin A1 (Figure 4A, 4B). On the contrary, increasing the expression of PRDX3 in RWPE-1 cells expressing low levels of PRDX3 led to a reduction in autophagy flux (Figure 4C, 4D). Therefore, PRDX3 is an autophagy inhibitor.

To further understand the mechanism by which PRDX3 inhibits autophagy, we investigate the impact of PRDX3 suppression on the levels of Beclin 1 and PI3KCIII, two interactive proteins regulating autophagy initiation [20, 21]. PRDX3 depletion did not alter the levels of Beclin 1 but led to an increase in levels of PI3KCIII (Figure 4E–4G), resulting in an activation of autophagy.

### Suppression of PRDX3 lead to suppression of pyroptosis

**Figure 5:**
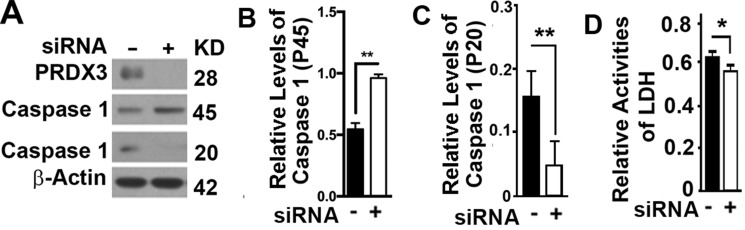
Impacts of PRDX3 on pyroptosis (**A**–**C**) Representative immunoblot (A) and quantification (B–C) showing the levels of caspase 1 (P45) (B) and caspase 1 (P20) (C) in BPH-1 cells treated with random (−) or PRDX3-specific siRNA (+). (**D**) Plots of lactate dehydrogenase (*LDH*) activity released in medium from cultured BPH-1 cells treated with random (−) or PRDX3-specific siRNA (+). Data are mean and standard deviation of three repeats and differences are tested with Student's *T*-test. *P ≤ 0.05; **P ≤ 0.01.

Autophagy directly regulates pyroptosis [22–24]. Reducing the expression of PRDX3 in BPH-1 cells led to an increase in the levels of caspase 1 (P45) and a reduction in the levels of Caspase 1 (P20) (Figure 5A–5C), suggesting an impairment of caspase 1 activation. Pyroptotic cells release lactate dehydrogenase (LDH) out of cell membrane [25]. Suppression of PRDX3 in BPH-1 cells resulted in a reduction in the released lactate dehydrogenase in the culture media (Figure 5D). Therefore, PRDX3 promotes pyroptosis.

## DISCUSSION

Eukaryotic mitochondrion is one of the most vital organelles resulted from symbiotic incorporation of α-proteobacteria into ancient archaea species. Although mitochondrion losses many functions during evolution, it gains a central role in the regulation of cell proliferation and death [26]. Mitochondria generate hydrogen peroxide through their respiratory chain and are the major sources of reactive oxygen species (ROS) in cells during normal metabolism or under pathological conditions [27]. PRDX3, the only mitochondrion-associated member of peroxiredoxin family, plays an important antioxidant role by catalyzing the conversion of hydrogen peroxide (H_2_O_2_) to H_2_O and helps maintain intracellular reactive oxygen species (ROS) steady state [9]. Active cell division and proliferation requires more energy that is produced by mitochondria so that high mitochondrial activity is induced and high mitochondria mass is accumulated. To remain a balance, high levels of PRDX3 evolves. It was reported that PRDX3 is overexpressed in prostate cancer and its overexpression promotes cancer cell survivals by protecting cells from oxidative stress [15, 28, 29]. The over-growth of prostatic cells characterizing BPH requires high mitochondrial mass and consequently high levels of PRDX3 as we observed in the BPH tissues.

Autophagy is the main pathway for cellular organelles and especially for mitochondria [30, 31]. It is induced under oxidative stress but simultaneously functions to mitigate oxidative stress [32]. For example, prostate stromal cells exhibit activated autophagy flux under hypoxia-induced oxidative stress [33]. On the one hand, PRDX3 depletion leads to oxidative stress enhancement that in turn autophagy activation. On the other hand, high levels of PRDX3 in epithelial cells in prostatic glands of BPH tissues are predicted to similarly inhibit autophagy flux. Autophagy promotes the clearance of damaged mitochondria that produce the reactive oxygen species. Autophagy inhibition leads to enhancement of oxidative stress and activation of pyroptosis. Epithelial cells died of pyroptosis will release pro-inflammatory stimuli to induce chronic inflammation and compensative growth of both epithelial cells and surrounding stromal cells shown in BPH tissues [12, 13, 34]. It was reported that activating the assembling of inflammasome led to production and secretion of IL-1β and IL-18 proinflammatory cytokines to perpetuate the inflammatory state associated with BPH [34, 35]. The compensative over-grown cells in BPH tissues have reached new balances so that levels of PRDX3 in the epithelial cells increase to counteract the elevated oxidative stress associated with increasing mitochondrial mass in the final stage of BPH development.

It seems contradicted that cells in tissues with high levels of PRDX3 exhibit high rates of growth but high levels of pyroptosis. It is logically postulated that cells with high levels of PRDX3 died of pyroptosis and disappeared under stresses so that the prostate tissues never became enlarged. However, due to the heterogeneity of cell population, some cells with sub-lethal amount of PRDX3 survive and may grow faster under chronic inflammatory stimulation induced by other cells died of pyroptosis. Those survived cells evolve to adopt the new environment in BPH tissues to express high levels of PRDX3 when the primary stresses that induce pyroptosis disappear. Those cells with high levels of PRDX3 in BPH tissues tend to commit pyroptosis to further promote inflammatory responses, compensative growth and potentially tumorigenesis. Therefore, PRDX3 inhibits autophagy flux and promotes pyroptosis to induce BPH.

## MATERIALS AND METHODS

### Antibodies, siRNAs, plasmids, and other reagents

Antibodies against human LC3 (catalog no. NB 100-2331) were purchased from Novus Biologicals. Primary antibodies against PRDX3 (catalog No.sc-59661), Beclin 1 (catalog no. sc-11427), and β-actin (catalog no. sc-47778), random sequence control siRNA (catalog no. sc-44234) and siRNA specific to PRDX3 (catalog no. sc-40833) were from Santa Cruz Biotechnology, Inc. HRP-conjugated secondary antibodies against mouse (catalog no. 172-1011) and rabbit (catalog no. 172-1019) were from Bio-Rad. FITC goat anti-rabbit IgG (catalog no. R6393 and A-21070), dihydroethidium (catalog no. D1168), Lipofectamine® 2000 (catalog no. 11668027), Oligofectamine (catalog no. 12252-011) and Pierce™ LDH Cytotoxicity Assay Kit (catalog no. 88953) were from ThermFisher Scientific. Antibody against caspase 1 (catalog no. PRS3459), and bafilomycin A1 were from Sigma-Aldrich. Antibody against TOM20 (catalog No. 612278) was purchased from BD Bioscience. Antibody against PI3KCIII (4263) was from Cell Signaling Technology.

### Enrollment of patients and collection of human prostatic tissue samples

This study was approved by the institutional review board of Guangzhou Medical University. The university provided the necessary institutional data and shared agreements before study initiation. A total 28 BPH patients were elected from those enrolled in the Guangzhou First People's Hospital affiliated with the university. A complete set of clinical data including age, serum PSA levels and prostate gland volume were collected. Normal prostate tissues were collected from 8 healthy donors. All collected prostatic tissues were processed and subjected to immunohistochemistry analyses as we previously reported [36, 37].

### Semiquantitative analysis

Immunostaining results presented as percentage of positively stained cells and staining intensity were estimated in a double-blinded manner similarly as described [36, 37]. Briefly, the percentage of positive cells was scored as 0 if no cells were stained, 1 if 1–10% cells stained, 2 if 11–50% cells stained, 3 if 51–80% cells stained and 4 if > 80% cells stained. The staining intensity was scored as 0 if no staining was seen, 1 if cells were weakly stained, 2 if they were moderately stained and 3 if they were strongly stained. The combined scores for intensity and frequency (0 to 7) represented the expression levels of PRDX3. The differences of staining scores of PRDX3 between different clinicopathological groups were evaluated with the Student's *T*-test.

### Culture of prostatic cells for immunochemistry analyses and assays of oxidative stress, lactate dehydrogenase activity and cell proliferation

Cell line BPH-1 developed from immortalized cells from human benign prostate hyperplasia with SV-40 large T-antigen and RWPE-1 (ATCC CRL-11609) derived from a histologically normal adult human prostate were cultured using standard techniques as described [38, 39]. The expression of PRDX3 in cultured prostatic cells were altered by transient transfection of either siRNAs or plasmids. Untreated or treated cells were fixed and subjected to fluorescent immunostaining with antibodies against PRDX3 and TOM20 for confocal microscopy analyses or harvested to prepare lysates for immunoblot analyses as we previously described [20]. Cultured cells were incubated with dihydroethidium to measure oxidative stress as we previously reported [40]. Culture media were collected and subjected to measuring the lactate dehydrogenase activity as we previously reported [23].

## References

[1] Berry SJ, Coffey DS, Walsh PC, Ewing LL (1984). The development of human benign prostatic hyperplasia with age. J Urol.

[2] Patel ND, Parsons JK (2014). Epidemiology and etiology of benign prostatic hyperplasia and bladder outlet obstruction. Indian J Urol.

[3] Emberton M, Fitzpatrick JM, Rees J (2011). Risk stratification for benign prostatic hyperplasia (BPH) treatment. BJU Int.

[4] Klionsky DJ, Abdelmohsen K, Abe A, Abedin MJ, Abeliovich H, Acevedo Arozena A, Adachi H, Adams CM, Adams PD, Adeli K, Adhihetty PJ, Adler SG, Agam G (2016). Guidelines for the use and interpretation of assays for monitoring autophagy (3rd edition). Autophagy.

[5] Mizushima N, Noda T, Yoshimori T, Tanaka Y, Ishii T, George MD, Klionsky DJ, Ohsumi M, Ohsumi Y (1998). A protein conjugation system essential for autophagy. Nature.

[6] Liu L, McKeehan WL, Wang F, Xie R (2012). MAP1S enhances autophagy to suppress tumorigenesis. Autophagy.

[7] Hornung V, Bauernfeind F, Halle A, Samstad EO, Kono H, Rock KL, Fitzgerald KA, Latz E (2008). Silica crystals and aluminum salts activate the NALP3 inflammasome through phagosomal destabilization. Nat Immunol.

[8] Lamkanfi M, Dixit VM (2014). Mechanisms and functions of inflammasomes. Cell.

[9] Ryter SW, Mizumura K, Choi AM (2014). The Impact of Autophagy on Cell Death Modalities. Int J Cell Biol.

[10] Yu J, Nagasu H, Murakami T, Hoang H, Broderick L, Hoffman HM, Horng T (2014). Inflammasome activation leads to Caspase-1-dependent mitochondrial damage and block of mitophagy. Proc Natl Acad Sci USA.

[11] Terlizzi M, Casolaro V, Pinto A, Sorrentino R (2014). Inflammasome: cancer's friend or foe?. Pharmacol Ther.

[12] Chughtai B, Lee R, Te A, Kaplan S (2011). Role of inflammation in benign prostatic hyperplasia. Rev Urol.

[13] St Sauver JL, Jacobson DJ, McGree ME, Girman CJ, Lieber MM, Jacobsen SJ (2008). Longitudinal association between prostatitis and development of benign prostatic hyperplasia. Urology.

[14] Lee S, Wi SM, Min Y, Lee KY (2016). Peroxiredoxin-3 Is Involved in Bactericidal Activity through the Regulation of Mitochondrial Reactive Oxygen Species. Immune Netw.

[15] Whitaker HC, Patel D, Howat WJ, Warren AY, Kay JD, Sangan T, Marioni JC, Mitchell J, Aldridge S, Luxton HJ, Massie C, Lynch AG, Neal DE (2013). Peroxiredoxin-3 is overexpressed in prostate cancer and promotes cancer cell survival by protecting cells from oxidative stress. Br J Cancer.

[16] He HC, Zhu JG, Chen XB, Chen SM, Han ZD, Dai QS, Ling XH, Fu X, Lin ZY, Deng YH, Qin GQ, Cai C, Chen JH, Zhong WD (2012). MicroRNA-23b downregulates peroxiredoxin III in human prostate cancer. FEBS let.

[17] Basu A, Banerjee H, Rojas H, Martinez SR, Roy S, Jia Z, Lilly MB, De León M, Casiano CA (2011). Differential expression of peroxiredoxins in prostate cancer: consistent upregulation of PRDX3 and PRDX4. Prostate.

[18] Lin WJ, Kuang HY (2014). Oxidative stress induces autophagy in response to multiple noxious stimuli in retinal ganglion cells. Autophagy.

[19] Mukhopadhyay SS, Leung KS, Hicks MJ, Hastings PJ, Youssoufian H, Plon SE (2006). Defective mitochondrial peroxiredoxin-3 results in sensitivity to oxidative stress in Fanconi anemia. J Cell Biol.

[20] Zou J, Yue F, Jiang X, Li W, Yi J, Liu L (2013). Mitochondrion-associated protein LRPPRC suppresses the initiation of basal levels of autophagy via enhancing Bcl-2 stability. Biochem J.

[21] Zeng X, Overmeyer JH, Maltese WA (2006). Functional specificity of the mammalian Beclin-Vps34 PI 3-kinase complex in macroautophagy versus endocytosis and lysosomal enzyme trafficking. J Cell Sci.

[22] Byrne BG, Dubuisson JF, Joshi AD, Persson JJ, Swanson MS (2013). Inflammasome components coordinate autophagy and pyroptosis as macrophage responses to infection. MBio.

[23] Chen Q, Yue F, Li W, Zou J, Xu T, Huang C, Zhang Y, Song K, Huang G, Xu G, Huang H, Li J, Liu L (2015). Potassium Bisperoxo(1,10-phenanthroline)oxovanadate (bpV(phen)) Induces Apoptosis and Pyroptosis and Disrupts the P62-HDAC6 Protein Interaction to Suppress the Acetylated Microtubule-dependent Degradation of Autophagosomes. J Biol Chem.

[24] Xu G, Yue F, Huang H, He Y, Li X, Zhao H, Su Z, Jiang X, Li W, Zou J, Chen Q, Liu L (2016). Defects in MAP1S-mediated autophagy turnover of fibronectin cause renal fibrosis. Aging (Albany NY).

[25] Rayamajhi M, Zhang Y, Miao EA (2013). Detection of pyroptosis by measuring released lactate dehydrogenase activity. Methods Mol Biol.

[26] Antico Arciuch VG, Elguero ME, Poderoso JJ, Carreras MC (2012). Mitochondrial regulation of cell cycle and proliferation. Antioxid Redox Signal.

[27] Chen Q, Vazquez EJ, Moghaddas S, Hoppel CL, Lesnefsky EJ (2003). Production of reactive oxygen species by mitochondria: central role of complex III. J Biol Chem.

[28] Shen C, Nathan C (2002). Nonredundant antioxidant defense by multiple two-cysteine peroxiredoxins in human prostate cancer cells. Mol Med.

[29] Chen L, Na R, Gu M, Salmon AB, Liu Y, Liang H, Qi W, Van Remmen H, Richardson A, Ran Q (2008). Reduction of mitochondrial H2O2 by overexpressing peroxiredoxin 3 improves glucose tolerance in mice. Aging Cell.

[30] Matsuda N, Tanaka K (2010). Uncovering the roles of PINK1 and parkin in mitophagy. Autophagy.

[31] Zou J, Yue F, Li W, Song K, Jiang X, Yi J, Liu L (2014). Autophagy inhibitor LRPPRC suppresses mitophagy through interaction with mitophagy initiator Parkin. PLoS One.

[32] Kongara S, Karantza V (2012). The interplay between autophagy and ROS in tumorigenesis. Front Oncol.

[33] Zhang N, Ji N, Jiang WM, Li ZY, Wang M, Wen JM, Li Y, Chen X, Chen JM (2015). Hypoxia-induced autophagy promotes human prostate stromal cells survival and ER-stress. Biochem Biophys Res Commun.

[34] Ponomareva L, Liu H, Duan X, Dickerson E, Shen H, Panchanathan R, Choubey D (2013). AIM2, an IFN-inducible cytosolic DNA sensor, in the development of benign prostate hyperplasia and prostate cancer. Mol Cancer Res.

[35] Kashyap M, Pore S, Wang Z, Gingrich J, Yoshimura N, Tyagi P (2015). Inflammasomes are important mediators of prostatic inflammation associated with BPH. J Inflamm (Lond).

[36] Jiang X, Li X, Huang H, Jiang F, Lin Z, He H, Chen Y, Yue F, Zou J, He Y, You P, Wang W, Yang W (2014). Elevated levels of mitochondrion-associated autophagy inhibitor LRPPRC are associated with poor prognosis in patients with prostate cancer. Cancer.

[37] Jiang X, Zhong W, Huang H, He H, Jiang F, Chen Y, Yue F, Zou J, Li X, He Y, You P, Yang W, Lai Y (2015). Autophagy defects suggested by low levels of autophagy activator MAP1S and high levels of autophagy inhibitor LRPPRC predict poor prognosis of prostate cancer patients. Mol Carcinog.

[38] Bello D, Webber MM, Kleinman HK, Wartinger DD, Rhim JS (1997). Androgen responsive adult human prostatic epithelial cell lines immortalized by human papillomavirus 18. Carcinogenesis.

[39] Niu Y, Ge R, Hu L, Diaz C, Wang Z, Wu CL, Olumi AF (2011). Reduced levels of 5-α reductase 2 in adult prostate tissue and implications for BPH therapy. Prostate.

[40] Li W, Zou J, Yue F, Song K, Chen Q, McKeehan WL, Wang F, Xu G, Huang H, Yi J, Liu L (2016). Defects in MAP1S-mediated autophagy cause reduction in mouse lifespans especially when fibronectin is overexpressed. Aging Cell.

